# Encephalopathy Associated with Autoimmune Thyroid Disease: A Potentially Reversible Condition

**DOI:** 10.1155/2016/9183979

**Published:** 2016-04-05

**Authors:** Inês Correia, Inês B. Marques, Rogério Ferreira, Lívia Sousa

**Affiliations:** ^1^Department of Neurology, Centro Hospitalar e Universitário de Coimbra, Praceta Professor Mota Pinto, 3000-075 Coimbra, Portugal; ^2^Department of Internal Medicine, Centro Hospitalar e Universitário de Coimbra, Praceta Professor Mota Pinto, 3000-075 Coimbra, Portugal

## Abstract

Autoimmune thyroid disease may occasionally associate with unspecific neurological symptoms, which are more commonly insidious, include cognitive or behavioural symptoms, and may associate with tremor, myoclonus, or ataxia. We report a 61-year-old female patient who presented with chronic headache, insidious mood, and cognitive disturbance which evolved in a few months to dementia associated with exuberant limb myoclonus. Diagnostic workup revealed high anti-thyroid peroxidase antibody titers and an inflammatory CSF profile, and it was negative for other possible etiologies. Treatment with steroids induced significant improvement. The diagnosis of encephalopathy associated with autoimmune thyroid disease is still controversial given the fact that the clinical presentation and diagnostic workup are unspecific, the pathophysiology is still undetermined, and the diagnosis is mostly of exclusion. No direct correlation is found between anti-thyroid antibody titers and clinical presentation, and it is currently speculated that other still unrecognized antibodies may be responsible for this clinical entity. It is extremely important to recognize this entity because it is potentially treatable with immunotherapies. It is also increasingly recognized that clinical improvement with first-line treatment with steroids may be absent or incomplete, and other immunotherapies as immunosuppressants, intravenous immunoglobulin, or plasma exchange must be attempted in the clinical suspicion of EEAT.

## 1. Introduction

Encephalopathy associated with autoimmune thyroid disease (EAAT) is a rare clinical entity, which presents with unspecific neurological symptoms. The clinical presentation is more frequently insidious, with cognitive and behavioural disturbance that may associate with tremor, myoclonus, or ataxia. More rarely, clinical onset may be acute as stroke-like episodes, epilepsy, or psychosis [1–6].

Diagnostic investigation is usually unspecific and there is no direct correlation between thyroid hormone levels or anti-thyroid antibody titers and the clinical presentation [1–3]. The current diagnostic criteria are based on the association of neurological or psychiatric symptoms, presence of anti-thyroid antibodies, exclusion of other possible causes, and significant improvement with immunotherapies [7], which make this entity mostly a diagnosis of exclusion.

We report a 61-year-old female patient who presented with chronic headache, insidious mood, and cognitive disturbance evolving to rapidly progressive dementia with exuberant limb myoclonus. Diagnostic workup identified high anti-thyroid antibody titers and excluded other causes, leading to diagnosis of EAAT. Significant improvement was achieved with steroid treatment.

With this paper we underline the importance of considering EAAT when approaching patients with rapidly progressive neurological or psychiatric symptoms, since it is a potentially reversible condition with appropriate treatment.

## 2. Case Report

We report a 61-year-old Caucasian woman who first presented to our Neurology Emergency Department in March 2012 complaining of severe chronic daily headache. The headache was started 9 months before and was described as bilateral, with pressing-type quality, without associated symptoms such as nausea, photophobia, or phonophobia, and did not worsen with recumbency, exercise, or Valsalva manoeuvres. The patient also presented apathy with progressive loss of interest in life for 18 months and had already been evaluated by a Psychiatrist who diagnosed depressive syndrome given that these features immediately followed the return to her country after 40 years living abroad, leaving behind her children and grandchildren. However, despite antidepressive treatment, she presented gradual worsening and became unable to perform her usual activities of daily living without supervision (such as cooking, using the telephone, handling money, and taking her medication) and she spent most days in bed in the last three months. The patient also complained of upper limb tremor with left predominance for the same period.

She had history of arterial hypertension, dyslipidemia, and hypothyroidism due to Hashimoto's thyroiditis chronically treated with levothyroxine. There was no history of recent or chronic infections or toxic exposure. Familial medical history was unremarkable.

The general physical examination was normal. The neurological examination revealed cognitive impairment with* Mini Mental State Examination* of 23 points (3 years of education). She presented frontal functions impairment with low verbal fluency, perseveration, impairment of abstract thinking, and signs of frontal release, namely, glabellar reflex. Visuospatial impairment was also observed with inability to copy a drawing or perform the clock-drawing test. An upper limb rest and postural tremor with left predominance was identified, without other focal signs in the neurological examination.

A brain computerized tomography (CT) and blood analysis were performed in the Emergency Department with normal results. On discharge a follow-up appointment was planned for dementia study. In the next weeks, a rapidly progressive neurological deterioration occurred; the patient became unable to walk and totally dependent and presented exuberant myoclonus in the distal upper limbs, so she was admitted for more investigations.

Extensive blood workup including full blood count, coagulation study, liver function test, creatinine, erythrocyte sedimentation rate, c-reactive protein, protein electrophoresis, vitamin B12, folic acid, thyroid function, and serologies (*Treponema pallidum*,* Brucella* spp.,* Borrelia burgdorferi*,* Coxiella burnetii*,* Rickettsia conorii*, HIV, and hepatitis B and C) was normal. Study of systemic autoimmunity, including antinuclear antibodies, anti-ds-DNA, anti-SSA, anti-SSB, anti-RNP, anti-Scl70, anti-Jo1, anti-neutrophil cytoplasmic antibodies, and anti-thyroid antibodies, revealed only high titers of anti-thyroid peroxidase antibodies (anti-TPO antibodies) equal to 1008 UI/mL (normal <40 UI/mL). Onconeural antibodies (anti-Hu, anti-Ri, anti-Yo, anti-amphiphysin, anti-Ma2, and anti-CV2) and tumoral markers (Carcinoembryonic Antigen, CA 19.9, CA 125, and CA 15.3) were negative. Antibodies against neuronal surface antigens (LGI-1 protein, NMDA AMPA, and GABA-B receptors) were also negative.

Cerebrospinal fluid (CSF) analysis revealed slightly increased proteins (72 mg/dL) and lymphocytic pleocytosis with 17 cells/mm^3^. CSF direct microscopy, cultures, and serologies (Herpes simplex 1 and 2, Cytomegalovirus, Epstein-Barr virus,* Treponema pallidum*,* Borrelia burgdorferi*, and* Brucella* spp.) were negative. Oligoclonal bands were absent and CSF dementia biomarkers (beta-amyloid peptide, tau protein, and phosphorylated tau protein) were normal.

Electroencephalogram (EEG) revealed rhythmic slow activity in both temporal regions, with normal background rhythm and without paroxysmal activity.

**Figure 1:**
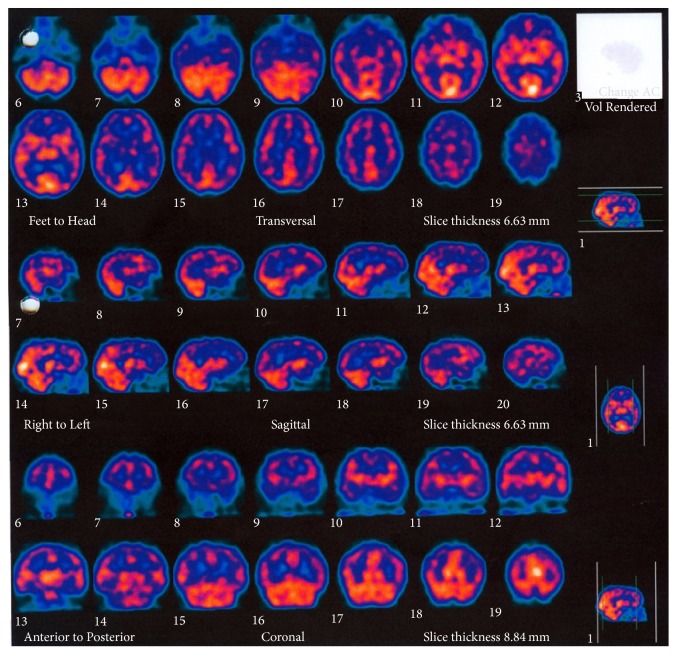
Brain perfusion SPECT. Reduced 99mTc-HMPAO uptake in parietal, temporal, and frontal lobes. Hypoperfusion is more severe on the left hemisphere.

Brain Magnetic Resonance Imaging (MRI) was unremarkable and brain perfusion single-photon emission computed tomography (SPECT) imaging revealed hypoperfusion in frontal, temporal, and parietal regions with left predominance (Figure 1).

The rapidly progressive neurological and psychiatric symptoms presented by the patient were unspecific and it could be the presentation of several different conditions including metabolic or toxic encephalopathy, CNS infection, cerebrovascular disease, CNS tumor, and CNS inflammatory conditions, such as cerebral vasculitis, autoimmune encephalitis, or paraneoplastic syndromes, or, more remotely, a rapidly progressive presentation of a degenerative dementia. The clinical history and diagnostic investigations excluded most of these causes and given that the only relevant findings were an inflammatory CSF profile and an increased anti-TPO antibodies titer, encephalopathy associated with autoimmune thyroid disease was diagnosed and treatment with intravenous methylprednisolone (1 g/day for 5 days) was performed. A significant improvement occurred after five days of therapy with complete resolution of mood and cognitive disturbance (MMSE = 29) and disappearance of the myoclonic movements. EEG was repeated with normal result. No steroid side effects occurred and, after improvement, the patient was discharged with oral prednisolone (1 mg/kg/day).

One month after discharge, steroid dosage reduction was attempted, but neurocognitive symptoms and distal limb myoclonus rapidly returned (anti-TPO antibodies = 217 UI/mL). Improvement was promptly seen after prednisolone reincrease to 1 mg/kg/day; however, steroid side effects developed, including Diabetes and Cushing Syndrome, so azathioprine was added as a steroid sparing agent. Although steroid gradual withdrawal was possible after 6 months without symptoms recurrence, the patient developed toxic hepatitis in relation to azathioprine and the drug was stopped. Neurological and psychiatric symptoms and myoclonic movements returned and were rapidly controlled with low prednisolone dosage (10 mg/day) without associated side effects, and other therapies such as plasma exchange, intravenous immunoglobulins, or other immunosuppressive drugs were not necessary. Three years later the patient was still asymptomatic, although anti-TPO antibody titer remains elevated (493 UI/mL). She is currently on prednisolone 5 mg/day as she is reluctant to discontinue for the risk of recurrence of symptoms.

**Figure 2:**
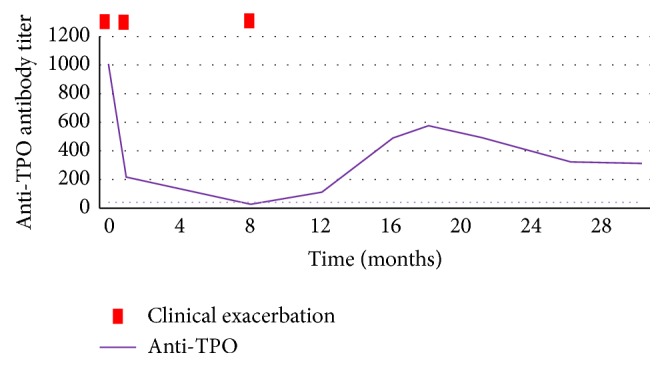
Evolution of anti-thyroid peroxidase antibodies (anti-TPO antibodies) titers in time and their relationship with clinical exacerbation.

**Figure 3:**
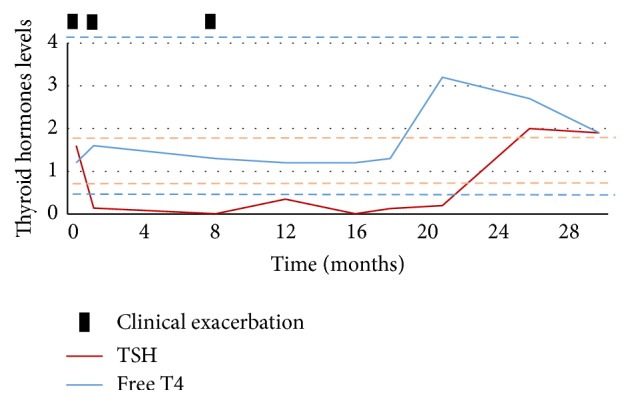
Evolution of thyroid hormone levels in time and their relationship with clinical exacerbation.

Figures 2 and 3 represent the evolution in time of anti-TPO antibody titers and thyroid hormones levels and their relation with clinical presentation. Although anti-TPO antibody titer is increased, there is no direct relationship with clinical presentation, including asymptomatic periods associated with elevated antibodies.

## 3. Discussion

Encephalopathy associated with autoimmune thyroid disease, also called Hashimoto's encephalopathy or Steroid-Responsive Encephalopathy associated with Autoimmune thyroid disease, is a controversial entity as its pathophysiology is not yet well defined and it is usually a diagnosis of exclusion. It is known to be associated with clinical or subclinical autoimmune thyroid disease, most commonly Hashimoto's thyroiditis, but there are also some reports of association with Graves Disease, a clinical entity without true thyroiditis [8–13].

Although autoimmune thyroiditis and the presence of anti-thyroid antibodies are relatively common in the population, with an estimated prevalence of Hashimoto thyroiditis of 0.3 to 2% [14–16] and detection of anti-thyroid antibodies in up to 10% healthy population [17–19], encephalopathy associated with thyroiditis or anti-thyroid antibodies is very uncommon, with an estimated prevalence of 2.1 per 100.000 habitants [20]. It occurs more commonly in females (4 : 1 ratio), and, although there are cases reported from childhood through the eighth decade of life, the mean age of onset is in the fourth decade [1, 2].

Since the first description of Hashimoto's encephalopathy in 1966 [21], the clinical spectrum has been widened and different possible presentations are now recognized, including acute-onset presentations as stroke-like episodes, epilepsy, or psychosis, and a slowly progressive presentation, which appears to be more common, characterized by cognitive and behavioral disturbances which may be associate with tremor, myoclonus, or ataxia [1–4].

The diagnostic criteria of EAAT include the association of neurological or psychiatric manifestations, high titers of anti-thyroid antibodies, exclusion of other possible causes with complementary exams, and a good response to immunosuppressive therapy [3, 7]. However, these vague diagnostic criteria may contribute to masquerade of other autoimmune encephalopathies instead.

In fact, blood workup is usually normal, except for the presence of increased anti-thyroid antibodies, more commonly against thyroid peroxidase (86%), but anti-thyroglobulin antibodies may be present in some cases (48%) [1–3]. Despite this association, there is no direct correlation between antibody titers and the clinical severity, with some asymptomatic patients having high antibody titers and some patients with severe encephalopathy having only mildly increased antibody titers. The absence of direct correlation between clinical presentation and anti-thyroid antibodies levels complicates the diagnosis and therefore the evidence of CNS inflammation, assessed in the cerebrospinal fluid (CSF), so the exclusion of other causes is essential to the assumption of this diagnosis. Thyroid hormones levels are also not related to the course of the disease with the majority of patients being euthyroid (18–45%) or hypothyroid (clinical in 25–35% and subclinical in 17–20%) and less commonly hyperthyroidism (7%) [1–3]. In fact thyroid hormone levels are usually normal or only mildly abnormal not to explain the psychiatric or neurological symptoms.

CSF analysis usually reveals mild and nonspecific inflammation that is mild mononuclear pleocytosis or slightly increased proteins [2, 13]; oligoclonal bands have been reported [6].

EEG abnormalities are usually present, occurring in 90 to 98% of patients, usually with unspecific findings [22]. The most common presentations are diffuse background slowing and frontal intermittent rhythmic delta activity (FIRDA) [1, 6], but other EEG patterns have been described such as periodic lateralized epileptiform discharges (PLEDs) [23] and temporal epileptiform activity [24, 25]. There is usually a correlation between the slowing severity and the encephalopathy severity [6, 24, 26] and an EEG normalization occurs with successful treatment, which may also be used to support the diagnosis [3, 27].

Brain MRI may be normal or, in up to 50% of cases, present unspecific anomalies, and it is very important to exclude other etiologies [1, 6]. The abnormalities that may be seen are cerebral atrophy, or less frequently, focal cortical abnormalities or unspecific focal or diffuse subcortical white matter hyperintensities which may be reversible with treatment [1, 3].

SPECT scan is not routinely used, but it was performed in some previous reports, with unspecific patterns of reduced perfusion, which can be diffuse or patchy and may involve multiple different regions [3, 28–30]. Positron emission tomography (PET) is also unspecific and usually shows widespread multifocal hypometabolism [31, 32]. The findings in SPECT and PET do not appear to be correlated with the clinical presentation, EEG, or neuroradiological findings and usually improve after successful treatment.

The pathogenesis underlying the encephalopathy associated with autoimmune thyroid disease is still unknown. Although anti-thyroid antibodies have no established pathogenic role, as there is no direct correlation between antibody titers and clinical severity [1–3], the high prevalence of coexistence of thyroid autoimmune diseases and other autoimmune diseases is well described [33, 34]. Therefore, the presence of anti-thyroid antibodies may be related to an autoimmune predisposition and the presence of other, still unrecognized, antibodies responsible for the encephalopathy may be speculated.

Antibodies against alpha-enolase, an antigen present in thyroid and also diffusely in the brain, have been described in some patients [35, 36]; however, their involvement in the pathogenesis has not been yet documented. The presence of antibodies against neuronal antigens is also suggested although it remains to be proven [37].

Given that EAAT is considered an inflammatory condition, the current treatment is based on immunotherapy. The most commonly used treatment is intravenous methylprednisolone (500–1000 g/day, for 3 to 5 days) followed by oral prednisone (1-2 mg/kg/day), which is gradually tapered with clinical improvement. Immunosuppressants, more commonly azathioprine, may be used as steroid sparing agents [3].

A prompt response to steroids occurs in most patients, usually with a favourable prognosis [3]. However, there is only partial benefit in some patients, no response to steroids is seen in a few cases, and, even after successful treatment, some patients may have relapses, usually during treatment withdrawal [3, 13, 25, 37–40]. However, the absence of response to steroids should not be regarded as a factor against this diagnosis, and other immunotherapies must be tried in cases of strong clinical suspicion. In cases of suboptimal or absent response to first-line treatment with steroids, good results have been reported with immunosuppressants (methotrexate, azathioprine, and cyclophosphamide), periodic intravenous immunoglobulin [41, 42], and plasma exchange [25]. More recently [43] a role for levetiracetam in patients ineligible to steroid treatment has been suggested, as having, in addition to its antiepileptic effects, a possible anti-inflammatory effect mediated through interleukin-1 beta and transforming growth factor beta 1.

In our case, both clinical presentation and response to treatment are in favour of an autoimmune encephalopathy and criteria to EAAT are met. However, both the absence of CSF oligoclonal bands and the correlation between blood antibodies and clinical exacerbation may favour the hypothesis of an autoimmune encephalopathy due to unknown antibodies, where anti-thyroid antibodies are just indicative of autoimmune predisposition.

In conclusion, encephalopathy associated with autoimmune thyroid disease is a diagnostic challenge as the clinical presentation and complementary exams are unspecific and no diagnostic markers are currently available. This condition is probably underdiagnosed in our clinical practice and, therefore, a high clinical suspicion is required. With this clinical case we underline the importance of making the diagnosis of this entity, since it can be treated with immunotherapy and patient prognosis can be significantly improved.

## Abbreviations

EAAT: Encephalopathy associated with autoimmune thyroid disease.

## Additional Points

- Clinical presentation of EAAT is variable and unspecific.
- Complementary exams results are unspecific and no diagnostic biomarker is available.
- There is no direct correlation between anti-thyroid antibody titers and the clinical presentation.
- The diagnosis consists mostly of exclusion of other possible causes.
- EAAT recognition is extremely important as it usually improves with immunotherapies.
